# Application of blood brain barrier models in pre-clinical assessment of glioblastoma-targeting CAR-T based immunotherapies

**DOI:** 10.1186/s12987-022-00342-y

**Published:** 2022-06-01

**Authors:** Jez Huang, Ying Betty Li, Claudie Charlebois, Tina Nguyen, Ziying Liu, Darin Bloemberg, Ahmed Zafer, Ewa Baumann, Caroline Sodja, Sonia Leclerc, Gwen Fewell, Qing Liu, Balabhaskar Prabhakarpandian, Scott McComb, Danica B. Stanimirovic, Anna Jezierski

**Affiliations:** 1 Human Health Therapeutics Research Centre, National Research Council of Canada, Building M-54, Montreal Road, ON K1A 0R6 Ottawa, Canada; 2 SynVivo Inc, Huntsville, AL USA 35806 701 McMillian Way NW,; 3 Biomedical Technology, CFD Research Corporation, Huntsville, AL USA 701 McMillian Way NW, 35806; 4 Department of Biochemistry, Microbiology and Immunology, Faculty of Medicine, University of Ottawa, Ottawa, ON Canada 451 Smyth Rd, K1H 8M5

## Abstract

Human blood brain barrier (BBB) models derived from induced pluripotent stem cells (iPSCs) have become an important tool for the discovery and preclinical evaluation of central nervous system (CNS) targeting cell and gene-based therapies. Chimeric antigen receptor (CAR)-T cell therapy is a revolutionary form of gene-modified cell-based immunotherapy with potential for targeting solid tumors, such as glioblastomas. Crossing the BBB is an important step in the systemic application of CAR-T therapy for the treatment of glioblastomas and other CNS malignancies. In addition, even CAR-T therapies targeting non-CNS antigens, such as the well-known CD19-CAR-T therapies, are known to trigger CNS side-effects including brain swelling due to BBB disruption. In this study, we used iPSC-derived brain endothelial-like cell (iBEC) transwell co-culture model to assess BBB extravasation of CAR-T based immunotherapies targeting U87MG human glioblastoma (GBM) cells overexpressing the tumor-specific mutated protein EGFRvIII (U87vIII). Two types of anti-EGFRvIII targeting CAR-T cells, with varying tonic signaling profiles (CAR-F263 and CAR-F269), and control Mock T cells were applied on the luminal side of BBB model in vitro. CAR-F263 and CAR-F269 T cells triggered a decrease in transendothelial electrical resistance (TEER) and an increase in BBB permeability. CAR-T cell extravasation and U87vIII cytotoxicity were assessed from the abluminal compartment using flow cytometry and Incucyte real-time viability imaging, respectively. A significant decrease in U87vIII cell viability was observed over 48 h, with the most robust cytotoxicity response observed for the constitutively activated CAR-F263. CAR-F269 T cells showed a similar cytotoxic profile but were approximately four fold less efficient at killing the U87vIII cells compared to CAR-F263, despite similar transmigration rates. Visualization of CAR-T cell extravasation across the BBB was further confirmed using BBTB-on-CHIP models. The described BBB assay was able to discriminate the cytotoxic efficacies of different EGFRvIII-CARs and provide a measure of potential alterations to BBB integrity. Collectively, we illustrate how BBB models in vitro can be a valuable tool in deciphering the mechanisms of CAR-T–induced BBB disruption, accompanying toxicity and effector function on post-barrier target cells.

**Supplementary Information:**

The online version contains supplementary material available at 10.1186/s12987-022-00342-y.

## Introduction

The recent success of chimeric antigen receptor (CAR)-T cell based immunotherapies for hematological malignancies has prompted interest in exploiting this cell based therapy for central nervous system (CNS) solid tumors [1]. CAR-T cells are T cells which are genetically engineered with an artificial receptor to target tumor specific-associated antigens, composed minimally of an antigen binding domain, a transmembrane domain, and one or more intracellular signaling domains. Most commonly, CAR-T cells are administered in autologous format, wherein T cells are collected from patients’ peripheral blood, expanded in vitro and genetically engineered to express CAR constructs. These modified CAR-T cells are then re-administered to the patient, where they target and lyse cells that carry the relevant tumor antigens [2].

Glioblastoma multiforme (GBM) is a highly aggressive and malignant brain cancer accounting for over 30% of primary CNS tumors with mean survival rates of 16–20 months [3, 4]. Due to the poor prognosis of patients treated with conventional therapies for GBM, attention has recently shifted to other emerging treatments, such as CAR-T based immunotherapies [1, 5–10]. The CNS, however, is an immune‐specialized organ presenting unique and specific challenges to the application of immunotherapy [6]. In contrast to blood cancers, the efficacy of immunotherapy for CNS tumors relies specifically upon the ability of the therapeutic immune cells to cross the blood brain barrier (BBB) to induce an anti-tumor response in the brain [6]. The BBB, formed by highly specialized brain endothelial cells, restricts the entry of substances larger than 600 Da [11] and naïve immune cells from the peripheral blood into the brain parenchyma. Under physiological conditions, immune cell trafficking into the CNS is tightly regulated by the BBB which selectively only allows entry of immune cell subsets required for immune surveillance [12]. The presence of neuroinflammatory conditions, which result in endothelial activation and upregulation of leukocyte adhesion molecules (such as ICAM and VCAM), facilitate naïve immune cells recruitment and trafficking across the BBB through a multistep cascade [13].

Another challenge that has impeded the development of CAR-T therapies for GBM is the limited availability of targetable tumor-specific antigens, which do not confer any risk of toxicity toward normal tissues. The mutant epidermal growth factor receptor variant III (EGFRvIII), is the most commonly observed EGFR variant in GBMs (30% of all GBMs), which is not expressed in healthy tissues, making it an ideal tumor specific antigen [14–16]. EGFRvIII arises from the deletion of exon 2–7 that leads to the generation of a novel glycine residue at the junction of exon 1 and 8 that creates a tumor-specific oncogenic and immunogenic moiety [14]. As a result, EGFRvIII targeting is of great therapeutic potential for antibody- and cell-based therapies. However, early phase clinical trials using systemically administered autologous EGFRvIII-CAR-T cells in patients with GBM have been met with limited success. Despite evidence of CAR-T cell trafficking into the brain parenchyma and infiltration at the tumor site with evidence of antigen decrease [17], clinical efficacy of EGFRvIII-CAR-T for GBM remains limited due low CAR-T life span, expansion and persistence as well as antigen loss, heterogeneity and adaptive changes in the tumor microenvironment [17–19]. There are currently several ongoing EGFRvIII-CAR-T clinical trials including combination therapies (reviewed in [20]).

To add to the complexity, CAR-T based therapies have also been shown to cause severe neurotoxicity. A subset of the patients undergoing CD19 CAR-T clinical trials for hematological malignancies, developed cytokine release syndrome (CRS) or immune effector cell-associated neurotoxicity syndrome (ICANS) [21–24]. The mechanisms of CAR-T induced neurotoxicity are not well understood, nor can they be reliably predicted. However, there is emerging evidence that the high levels of systemic inflammatory cytokines (IL6, TNFγ and TNFβ) lead to endothelial cell activation and BBB disruption resulting in increased BBB-permeability and peripheral cytokine and immune cell infiltration into the CNS [21–27]. This subsequently initiates a feedback loop of continued endothelial activation perpetuating neurotoxicity events. Some data has suggested that CD19 expression in the brain might also drive neurotoxicity [28], but recent observation of ICANS in a prostate-specific membrane antigen (PSMA)-targeted CAR-T clinical trial may be suggestive that neurotoxicity is antigen-independent [29]. This has highlighted the importance of optimization of CAR constructs, as well as the need for early preclinical modeling of BBB disruption and neurotoxicity, for systemically administered CAR-T therapies [30]. Since crossing the BBB is an important step to the success of systemic applications of CNS targeting CAR-T based therapies, we sought to evaluate the BBB extravasation, disruption and cytotoxic effector function post-BBB of two EGFRvIII-targeting CAR-T candidates in an iPSC-derived BBB model.

## Methods

### U87vIII cell culture

U87MG cells expressing EGFRvIII (U87vIII) via retroviral transduction and sorting were kindly provided by Professor Cavnee, from the Ludwig Institute for Cancer Research, University of California, San Diego (San Diego, CA, USA) [31, 32]. To more easily visualize target U87vIII cells in cytotoxicity assays, stable lines expressing nuclear-localized mKate2 (U87vIII-mKate2) were generated using commercially obtained lentivirus (Lenti Nuclight-Red, Incucyte, Sartorius). U87vIII and U87vIII-mKate2 cells were cultured in poly-L-lysine coated T-75 flasks containing Dulbecco’s modified Eagle’s (DMEM) medium supplemented with 10% (vol/vol) heat-inactivated fetal bovine serum (FBS) (Hyclone), 50 U/ml penicillin, 50 U/ml streptomycin, 2 mM L-glutamine, and 0.2 mg/ml G-418 (all from Life Technologies) at 37 °C with 5% CO_2_. Complete media was changed every 3 day.

### CAR-T transduction

Primary human T cells were isolated from whole blood obtained from healthy human volunteers under informed consent and approval through the National Research Council of Canada Research Ethics Board. In brief, T cells were isolated from peripheral blood mononuclear cells (PBMCs) freshly isolated from healthy blood donors via negative magnetic selection. The T cells were activated with MACS GMP TransAct CD3/CD28 beads (Miltenyi) cultured in ImmunoCult XF media (Stem Cell Technologies) supplemented with 20 U/ml IL-2 (Proleukin, Novartis). Activated T cells were typically transduced with CAR-GFP lentiviral vector (F263-28z and F269-28z, as described in [33]), 24 h post-stimulation and expanded in IL2-supplemented expansion media (20 IU/ml) with strict maintenance of cell concentrations below 5 × 10^5^ cells/ml. All cell counting was performed using an automated cell counter (Cellometer; Nexcelcom) to assess live/dead counts using acridine orange/propidium iodide (PI) staining. Efficiency of transduction was assessed at day 7 by flow cytometry and at day 10 the CAR-F263 and CAR-F269 T cells were used for assays. Mock T cells underwent the same treatment as CAR-transduced T cells but without virus infection. Cell acquisition was performed using a BD- Fortessa (BD Biosciences). Post-acquisition analysis was performed using FlowJo software.

### Differentiation of iPSCs into brain endothelial-like cells (iBECs)

All experimental protocols using human amniotic fluid derived induced pluripotent stem cells (AF-iPSCs) were performed following the guidelines established and approved by the National Research Council Canada Research Ethics Board and the in accordance with relevant guidelines and regulations as approved by the Ottawa Hospital Research Ethics Board. AF-iPSC were generated from human amniotic fluid (AF) cells and differentiated into iBECs, as previously described [34, 35]. In brief, AF-iPSC were seeded at a density 8 × 10^3^ cells/cm^2^ in DMEM/F12 medium (Life Technologies) supplemented with 20% KnockOut Serum Replacement, 1 × Glutamax, 1 × Non-Essential Amino Acids, and 0.1 mM β-mercaptoethanol (all from Life Technologies) for 6 days. The medium was changed to EM medium (human Endothelial Serum-Free medium, Life Technologies) supplemented with 20 ng/ml basic fibroblast growth factor (bFGF, Life Technologies), 10 μM retinoic acid (RA, Sigma) and 1% fetal bovine serum (FBS, Hyclone) for an additional 2 days. To establish the in vitro transwell BBB model, iBECs were dissociated with Accutase (Stem Cell Technologies) and seeded at density of 2.5 × 10^5^ cells per 24 well transwell insert (3 µm pore size, 0.33 cm^2^ surface area; BD Falcon) pre-coated with collagen type-IV (80 µg/ml, Sigma) and fibronectin (20 µg/ml, Sigma) in complete EM medium with 10 µM Y27362 (ROCK Inhibitor, Stem Cell Technologies), as previously described [34]. iBECs transwells were incubated overnight at 37 °C in 5% CO_2_ and the next day the medium was changed to EM medium without bFGF and RA for an additional 24 h in the luminal chamber.

### Trans-endothelial electrical resistance (TEER) measurements

After 2 days post-seeding on the transwell inserts, transendothelial electrical resistance (TEER) was assessed prior to performing BBB extravasation, sodium fluorescein (NaFl) permeability and U87vIII pre-conditioning assays. A CellZscope apparatus (NanoAnalytics) was used to conduct the TEER measurement. The values are normalized by subtracting the background (TEER of the empty inserts) and reported in Ω·cm^2^, as previous described [35].

### BBB extravasation and cytotoxicity assay

A day prior to the BBB extravasation assays, 5 × 10^4^ U87vIII-mKate2 cells were plated onto a poly-L-lysine coated 24-well companion plates (BD Falcon). Approximately 24 h post-plating, the 24-well transwell iBECs inserts were pre-incubated with U87vIII-mKate2 cells in EM media for 2 h prior to the addition of the CAR-T/T cells into the luminal chamber. Following iBEC-U87vIII-mKate2 cell preconditioning, 250 µl of EM medium was removed from the luminal side of the inserts and 250 µl containing 2.5 × 10^5^ Mock, CAR-F263 or CAR-F269 T cells in EM medium were added to the inserts and placed into the Incucyte S3 Live Cell Analysis System (Sartorius). Continuous live-cell imaging was used to assess U87vIII-mKate 2 cytotoxicity in the abluminal chamber over 48 h post addition of the CAR-T/T cells. After proper image calibration, the Incucyte software package allows phase and red object counts and area assessments, enabling determination of U87vIII-mKate2 positive target cell confluency in the visual field as a functional measure of CAR-T/T cell mediated cytotoxicity post-BBB extravasation. Images in the abluminal companion plate were acquired every 2 h using phase contrast as well as red (ex., 565–605 nm; em., 625–705 nm) fluorescent channels for up to 48 h. Sixteen images were taken from each well and the confluency percentage data and red objectives count were recorded at the same time. The value reported, per treatment condition, was the mean of 16 images per well.

### Flow cytometry

Flow cytometry was used to quantify the number of CAR-T/T cell extravasation across the BBB at 3, 6 and 24 h time points. At each time point, 100 µl of EM medium was removed from the bottom chamber of the companion plate for analysis. Following each collection, 100 µl pre-warmed EM medium were added back to the wells. The CAR-T/T cells were incubated with anti-CD45 (BD Biosciences) and anti-CD25 (BD Biosciences) diluted in staining solution (1:1 mixture of Brilliant Stain Buffer Plus (BD Biosciences) and PBS with 1% FBS, 10 mM HEPES, and 2 mM EDTA) for 30 min at room temperature, centrifuged, re-suspended in fixation solution (1% formaldehyde in PBS) and immediately analyzed using the BD Fortessa flow cytometer. Forward- and side-scatter and CD45 positive signal, based on unstained controls, were used to gate on T cells, respectively. Forward-scatter height vs. forward-scatter area was used to gate on single cells. Analysis was performed using FlowJo software.

### BBB permeability assays

To evaluate whether CAR-T/T cells had an effect on the permeability of the iBEC monolayer, NaFl permeability (Pe) (luminal to abluminal) was performed after 24 h. Briefly, the iBEC transwell inserts were washed with 1 ml 1 × Hank’s buffered saline solution (HBSS) (Wisent) to remove residual T cells and medium. The inserts were then placed into plates with 1 ml of transport buffer (5 mM MgCl_2_ and 10 mM HEPES in HBSS, pH 7.4) and incubated at 37 °C for 10 min and then 250 µl of the transport buffer was removed from the luminal chamber of each insert and replaced with 250 µl of NaFl (50 µg/ml) in transport buffer. The plates were then incubated at 37 °C with gentle rotation (20 rpm/min) and 100 µl of transport buffer was collected from the bottom of the wells at 15, 30, 45 and 60 min intervals for permeability analysis; 100 µl transport buffer were added back to the wells and the plates were returned to the incubator. Inserts without iBEC were used for the background controls. The quantitation of NaFl was performed using a fluorescent plate reader (ex., 485 nm and em., 530 nm) and plotted against a standard curve (0–50 ng NaFl solution in transport buffer), as previous described [35].

### iBEC activation assay

iBECs were seeded at density of 1 × 10^6^ cells/cm^2^ on a 24 well plate that were pre-coated with collagen type-IV (80 µg/ml, Sigma) and fibronectin (20 µg/mL, Sigma) in complete EM medium with 10 µM Y27362 (ROCK Inhibitor, Stem Cell Technologies). iBECs were stimulated with 300 ng/ml of recombinant human TNF-α (R&D Systems) and 200 IU/ml recombinant human IFN-γ (R&D systems) for 24 h. For GBM co-culture experiments, 5 × 10^4^ U87vIII cells were plated on a 24-well plate and 2.5 × 10^5^ iBECs were seeded on 24 well transwell inserts and co-cultured for 12–24 h. Stimulated (GBM co-culture vs cytokine) and non-stimulated control cells were gently detached with Accutase (Stem Cell Technologies) and washed with 1% bovine serum albumin (BSA, Sigma)/ PBS (Wisent Bioproducts). Cells were blocked with anti-CD16/CD32 monoclonal antibody (1:100, Thermo Fisher Scientific) for 10 min. Cells were stained with conjugated antibodies (Additional file 4: Table S1) for 30 min at room temperature and then washed with 1% BSA/PBS. Cells were acquired with the BD Accuri C6 Plus flow cytometer (BD Biosciences). Forward- and side-scatter on unstained control were used to gate on cells, respectively. Forward-scatter height vs. forward-scatter area was used to gate on single cells. Analysis was performed using FlowJo software.

### SynVivo BBB-on-CHIP (SynBBB)

The SynBBB device is comprised of a 200 µm wide outer channel separated by a central chamber of 500 µm width in communication across utilizing microfabricated pores of 3 μm similar in size to the transwell membranes. All the chambers are 100 μm height. All channels were first coated with collagen/fibronectin solution with a concentration of 200 µg/ml fibronectin (Sigma Aldrich) and 800 µg/ml collagen type-IV (Sigma Aldrich) at room temperature. The chip was primed for 20 min using a pneumatic primer (SynVivo) purging nitrogen gas at 7 psi and placed in the incubator at 37 °C for at least 1 h until use. Before seeding the chips with iBECs, all the channels were flushed with EM medium.

iBECs were seeded in EM medium with 10 µM Y27362 (ROCK Inhibitor, Stem Cell Technologies) into the endothelial channel at a concentration of 2.5 × 10^7^ cells/ml via Tygon tubing. As the cell suspension was infused through the channels, optical microscopy was used to visualize cell distribution. Once the iBECs fully occupied the central channel, the chip was placed in the incubator overnight to allow the cells to settle and attach to the bottom surface. After the iBECs fully attached to the bottom surface, the channel was connected to a 1 ml syringe containing complete EM medium and a programmable syringe pump (PHD Ultra, Harvard Apparatus) was used to push media through the channel, removing unattached cells while changing media. The media flow rate was ramped from 0.01 µl/min to 1 µl/min over the next 24 h to exercise the cells to physiologically relevant shear stress. Thereafter, media flow rate was maintained at 1 µl/min until the iBECs form a 3D lumen structure.

On day 2, U87vIII were seeded to the side channels at 1 × 10^6^ cells/ml, media was changed every 12 h at 0.5 µl/min for 10 min. On day 3, iBECs were confluent and formed a 3D barrier lumens. Prior to infusing the CAR-F263 cells, barrier integrity was assessed by perfusing NaFl (25 µg/ml) through the endothelial channel at 0.2 µl/min. The fluorescent images were acquired after 15 min perfusion (before and after CAR-T extravasation assay) to determine the ratio of fluorescence in the tissue vs. endothelial channel. The images were analyzed using ImageJ rectangle selection tool to select an area (> 200 µm × 200 µm) within the channel. Measurement parameter was set to analyze the mean gray value of the area and repeated measurements were performed on six random regions within each channel to obtain average intensity values. The intensity ratio between the tissue and endothelial channel were calculated and ratio values below 0.2 were used as a quality control cut-off value to indicated intact barrier formation. Once the iBEC barrier was formed, CAR-F263 cells were labeled with 0.33 µM Incucyte^®^ Cytolight Rapid Green Dye (Sartorius) for 30 min and infused 0.5 µl/min for 10 min for CAR-F263 T cell accumulation followed by 0.1 µl/min for 24 h for constant flow condition. Phase contrast images and fluorescent images (red channel, ex., 565–605 nm; em., 625–705 nm) were acquired every 30 s and the movies were played at 4 frames per second.

### RNASeq analysis

Total RNA was extracted from cell pellets using NucleoSpin RNA plus kit (Macherey–Nagel GmbH & Co. KG) according to manufacturer’s instructions. Genomic DNA contamination was removed by Turbo DNA-Free Kit (Life Technologies). RNA quality was assessed using Agilent Bioanalyzer 2100. RNASeq Libraries were generated using the TruSeq strand RNA kit (Illumina). The libraries were quantified by Qbit and qPCR according to the Illumina Sequencing Library qPCR Quantification Guide and the quality of the libraries was evaluated on Agilent Bioanalyzer 2100 using the Agilent DNA-100 chip. The RNASeq library sequencing was performed using Illumina Next-Seq500. STAR (v2.5.3a) [36] was used for alignment of the reads to the reference genome and to generate gene-level read counts. Human (Homo sapiens) reference genome (version GRCh38.p13) [37] and corresponding annotation were obtained from Gencode (https://www.gencodegenes.org/human/release_33.html) and used as reference for RNASeq data alignment process. DESeq2 [38] was used for data normalization. The expression value of each gene was expressed, as average read counts, of three replicates.

## Results

### Endothelial cell activation in iBECs

**Fig. 1:**
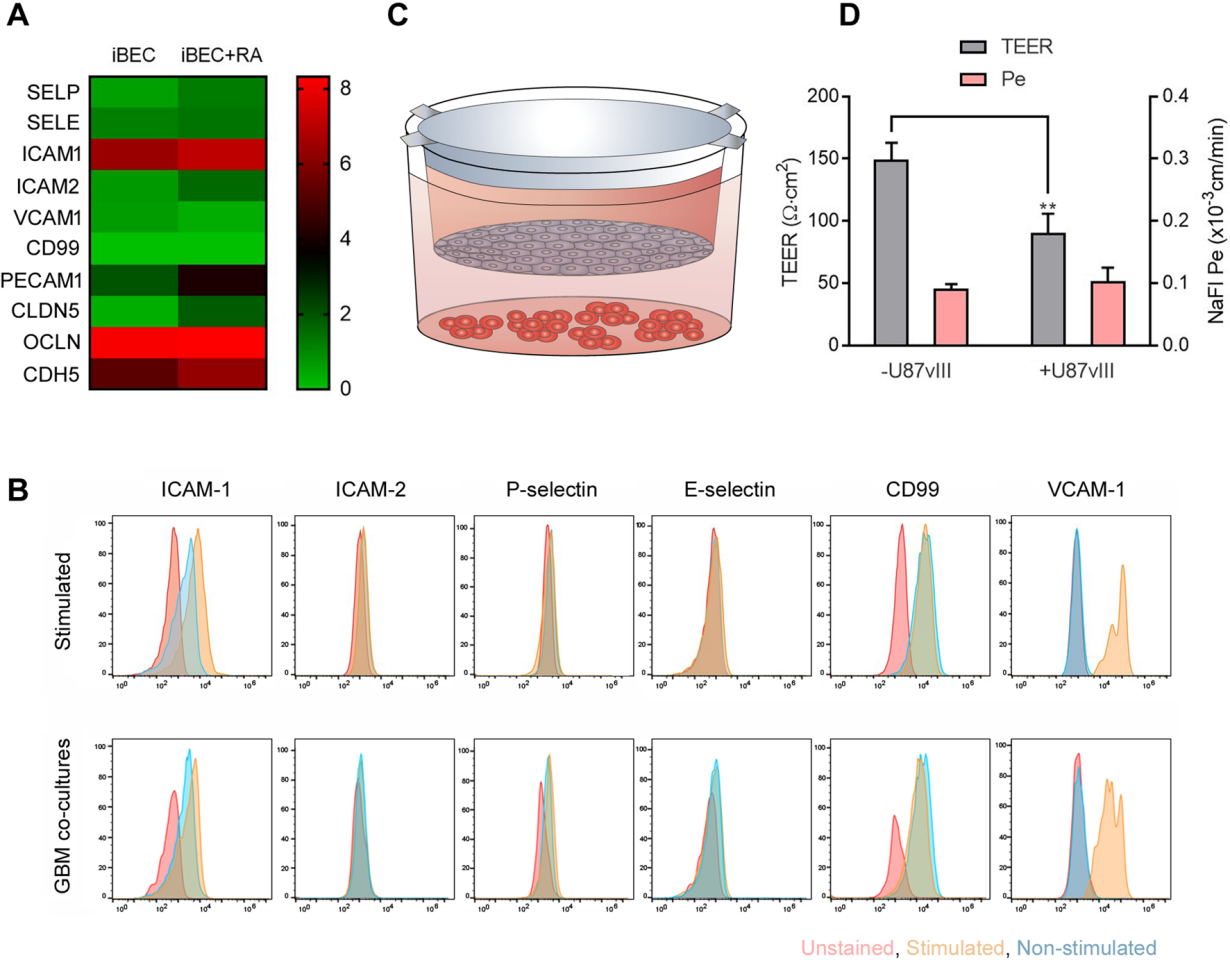
Expression of immune cell adhesion molecules on iBECs. **A** Heatmaps depicting log2 transformed transcript abundances of the immune cell adhesion and BBB-specific genes in iBEC in the absence and presence of Retinoic Acid (+ RA) treatment. *Green* low-expression, *Red* high- expression. **B** Cell surface analysis for expression of adhesion molecules ICAM-1, ICAM-2, P-selectin, S-selectin, CD99 and VCAM1 in iBECs using flow cytometry under naïve (non-stimulated—blue) and cytokine (top panel) and GBM co-culture (bottom panel) stimulated (yellow) conditions. Red—unstained controls. See Additional file 4: Table S2 for MFI values. Representative data shown from 2 independent differentiations. **C** Schematic illustrating set up of transwell blood–brain barrier (BBB) and U87vIII co-culture model. **D** Assessment of TEER (Ω cm^2^) following 24 h co-culture of iBEC transwell inserts with ( +) and without (−) U87vIII cells. TEER and NaFl Pe values are expressed as mean + standard deviation (SD). Statistical significance marked by asterisks assessed by Student T-test where **P ≤ 0.01 (n = 3)

In this study, we used the well-established human iPSC-derived brain endothelial-like cell (iBEC) transwell model (previously described in [35]) to assess endothelial cell activation and CAR-T cell extravasation. Under physiological conditions, iBECs express low levels of immune cell adhesion genes, as assessed by RNASeq analysis, with the exception of *ICAM-1* which shows robust basal expression (Fig. 1a). Retinoic acid (RA) treatment, during iBEC differentiation, induced expression of immune cell adhesion as well as BBB-related genes (Fig. 1a), as previously described [39]. Stimulation with a combination of pro-inflammatory cytokines (100 ng TNFα, 200 IU/ml INFγ and 200 ng/ml TNFα as well as 300 ng/ml of TNFα alone), induced the expression of VCAM-1, ICAM-1 and CD99; however, no significant upregulation for ICAM-2 or P- and S-selectins was observed (Fig. 1b, top panel; Additional file 1: Figure S1). This immune phenotype is consistent with previous reports for other human iPSC-derived iBECs [40–42]. When iBECs were co-cultured with human glioblastoma (GBM) overexpressing EGFRvIII (U87vIII) cells (Fig. 1c), a similar immune adhesion marker profile to cytokine stimulated iBECs was observed (Fig. 1b, bottom panel). In addition to inducing iBEC activation (Fig. 1b), U87vIII co-cultures also led to a significant decrease in TEER and changes in sodium fluorescein (NaFl) permeability (Fig. 1d). These findings align with previous reports of decreased TEER following exposure to immune cytokines and with clinical evidence of the disruption of BBB integrity in GBM tumors [43, 44]. Of note, as TEER values are influenced by the insert surface area and pore size [45], we routinely observe lower TEER values using 24 well inserts with 3 µm pore size (~ 150 Ω cm^2^) vs 12 well inserts with 1 µm pores (~ 300–500 Ω cm^2^)[35]. Since endothelial ICAM-1 and VCAM-1 expression preferentially promotes leukocyte adhesion and facilitates early steps in leukocyte extravasation across brain endothelial cells [46–48], we next used this model to assess CAR-T cell extravasation and post-BBB cytotoxicity on U87vIII cells in a blood–brain-tumor barrier (BBTB) transwell model.

### CAR-T extravasation across the BBB

**Fig. 2:**
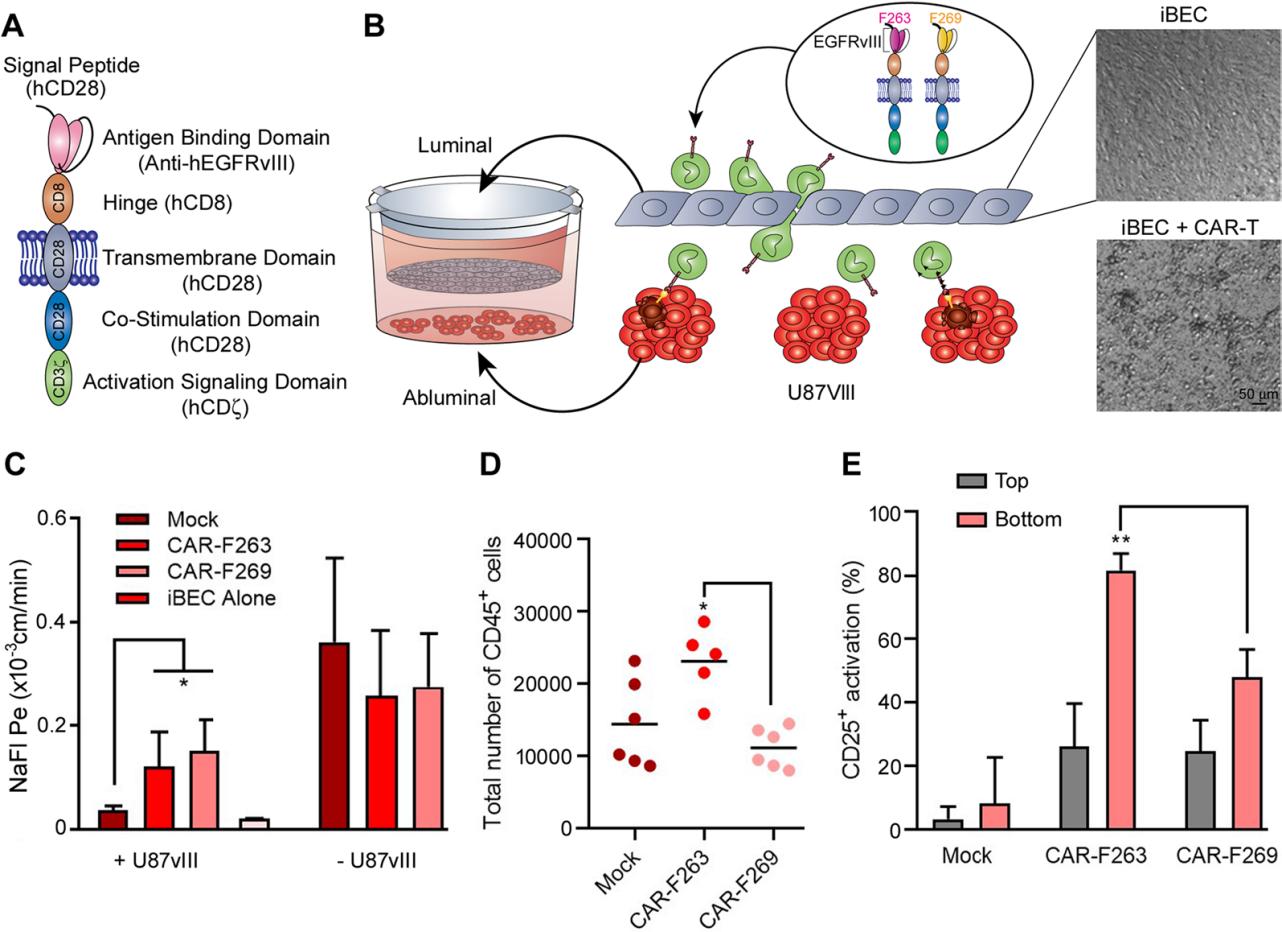
CAR-T extravasation across the BBB. **A** Schematic of single-chain variable fragment (scFV) structure of EGFRvIII-CAR. **B** Schematic illustrating set up of transwell BBB and U87vIII co-culture system to establish blood–brain tumor barrier (BBTB) model. Phase contrast images in the luminal chamber showing CAR-T cell interacting with the iBEC monolayer in the transwell inserts. Scale bar = 50 µm (**C**) Assessment of BBB integrity using sodium fluorescein (NaFl) permeability (Pe), 24 h following addition of CAR-T cells (CAR-263 and CAR-269) and Mock T cells to iBEC transwell cultures in the presence or absence of U87vIII cells in the companion plates. Pe is expressed as the mean + SD. **D** Quantification of CAR-T/T cells extravasation in the abluminal chamber of the companion plate after 24 h based on CD45 expression assessed by flow cytometry. **E** Quantification of CAR-T/T cell activation, in both top (luminal) and bottom (abluminal) compartments based on CD25 expression, as a percentage of CAR-transduced GFP expressing cells, assessed by flow cytometry. Data presented as mean + SD and statistical significance is marked by asterisks assessed by one-way analysis of variance (ANOVA) where *P ≤ 0.05, ** ≤ 0.01 (n = 5)

To assess CAR-T cell extravasation across the iBEC monolayers, anti-EGFRvIII CAR-T cells with two different single-chain variable fragments were used in this study: CAR-F263 and CAR-F269 (Fig. 2a), as previously described [33]. While both EGFRvIII-CAR-T molecules show strong on-target activity against EGFRvIII, CAR-F263 cells show a higher basal activation state (tonic signaling)[33]. As control cells, we also used unmodified Mock T cells handled in a mock transduction protocol in the absence of lentiviral vector (no CAR). To ensure that there was no off-target effects on the iBECs, the CAR-T and Mock T cells were first co-cultured with the iBECs for 24 h and no iBEC cell death was observed (Additional file 2: Figure S2). CAR-F263, CAR-F269 and Mock T cells were subsequently added to the luminal (top) compartment of the iBEC transwells (Fig. 2b) either in the presence or absence of U87vIII cells in the abluminal (bottom) compartment of the model. The CAR-T cells remained viable during the course of the experiment and adhered and interacted with the iBEC monolayer in the insert (Fig. 2b, phase contrast image). Barrier integrity was assessed, after 24 h co-culture, using NaFl permeability assay. In the presence of U87vIII cells, we observed an increase in permeability for Mock as well as CAR-F263 and CAR-F269 T cells (Fig. 2c, left). As this represents an additive effect on BBB disruption (U87vIII and CAR-T/T cells), we also examined permeability in the absence of U87vIII cells to discriminate CAR-specific disruption of the BBB. Compared to Mock T cells and iBECs alone, CAR- F263 and CAR-F269 significantly increased BBB permeability (Fig. 2c, right).

We subsequently quantified CAR-T cell extravasation after 24 h, in the presence of U87vIII cells in the abluminal chamber, by flow cytometry analysis based on CD45 expression. Approximately 5–10% of the input 2.5 × 10^5^ cells were detected in the bottom chamber, with higher amounts of the constitutively active CAR-F263 (23 106 ± 4 788; 9%) compared to CAR-F269 (11 150 ± 2 758; 4.5%) and Mock T cells (14 422 ± 6 076; 5.7%) (Fig. 2d). We further assessed T cell activation by examining CD25 expression, as a percentage of CAR (GFP^+^) expressing T cells (or total T cells for mock), in the luminal and abluminal compartments (Fig. 2e). The tonic signaling CAR-F263 showed higher activation compared to CAR-F269 in the abluminal chamber, with both CAR-F263 and CAR-F269 activation being significantly higher than Mock T cells. Interestingly, activation profiles in the luminal compartment were similar for both CAR-F263 and CAR-F269 and higher compared to Mock T cells. This activation state of both CAR-F263 and CAR-F269 may be due to interaction with iBEC cells and could explain the observed disruption in the BBB integrity. Overall, these data indicate that tonic signaling CAR-T cells show elevated BBB extravasation in this model.

### CAR-T mediated cytotoxicity of U87vIII cells

**Fig. 3:**
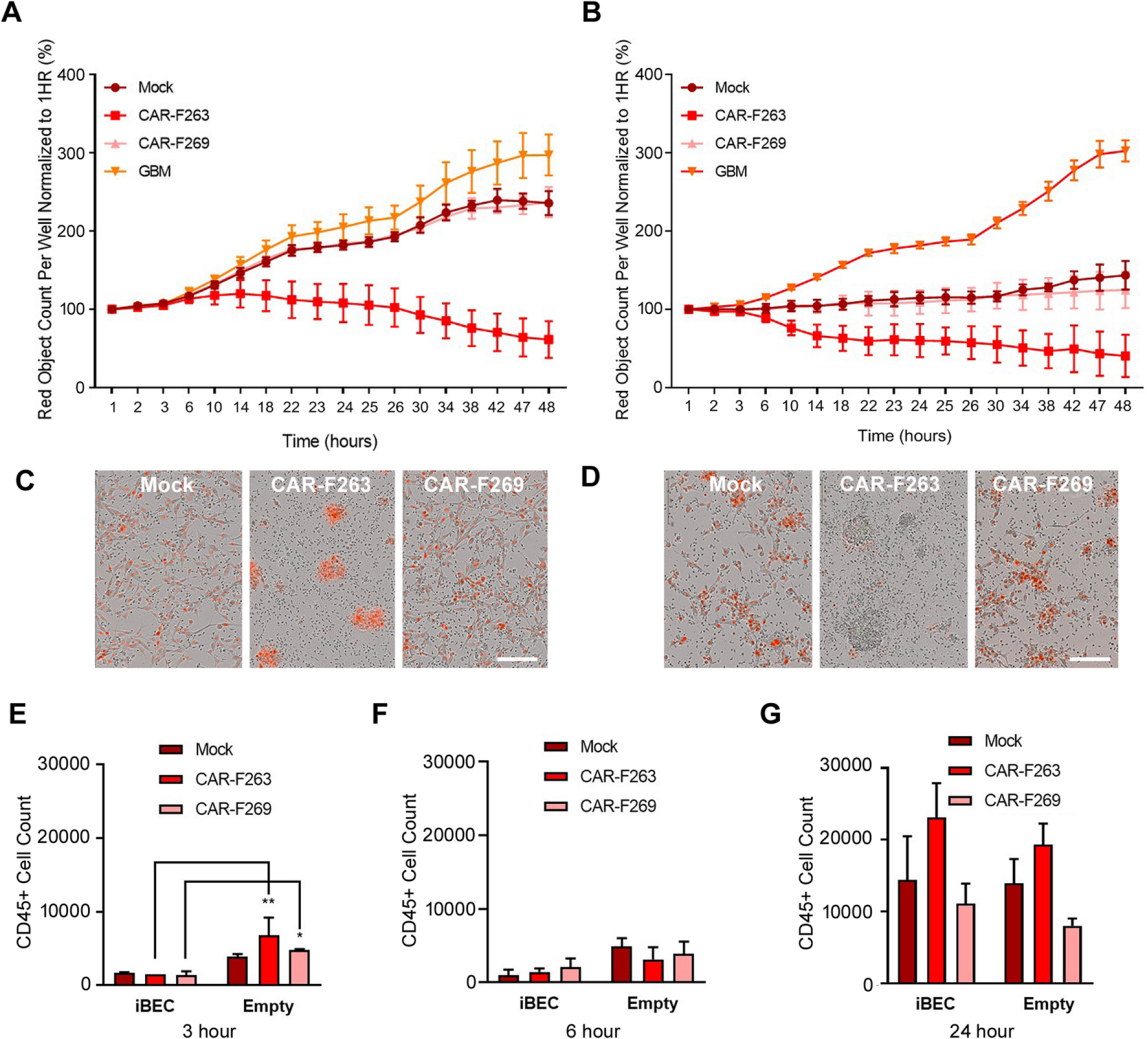
CAR-T mediated cytotoxicity of U87vIII-mKate2 target cells. **A** Post-BBB anti-EGFRvIII CAR-T effector function of CAR-F263, CAR-F269 and Mock T cells, in transwells contained iBECs, assessed in the abluminal compartment using continuous live-cell imaging (Incucyte) of U87vIII-mKate2 cells (red) proliferation as a measure of CAR-mediated killing. Data shown presented as percentage (%) of red object count per well. **B** anti-EGFRvIII CAR-T effector function of CAR-F263, CAR-F269 and Mock T cells, in empty inserts, assessed in the abluminal compartment using continuous live-cell imaging (Incucyte) of U87vIII-mKate2 proliferation as a measure of CAR-mediated killing. Data shown presented as percentage (%) or red object count per well. Representative data form three independent experiments. **C–D** Representative images of U87vIII-mKate2 killing in the albuminal chamber, for **A** and **B** respectively, after 48 h. Scale bar = 100 µm. See Additional file 3: Movie S1 for real-time movie showing CAR-F263, CAR-F269 and Mock T cell mediated U87vIII-mKate2 killing in the abluminal chamber. Quantification of CAR-T/T cell extravasation (mean + SD), across the BBB and empty inserts, in the presence and absence of U87vIII cultures in the companion plates assessed by CD45^+^ expression using flow cytometry at (**E**) 3, (**F**) 6 and (**G**) 24 h. Statistical significance marked by asterisks assessed by one-way analysis of variance (ANOVA) where *P ≤ 0.05

To examine the CAR-T cell effector functions within the abluminal chamber, we used automated live cell imaging of co-cultures of U87vIII cells stably expressing nuclear-localizes mKate2 (U87vIII-mKate2, red) in the companion plates (Fig. 3a). A significant decrease in U87vIII cell viability was observed, over the 48 h time course, with a faster and more robust U87vIII killing response observed for CAR-F263 (Fig. 3a, c). CAR-F269 and Mock T cells showed a similar killing profile but approximately four fold less efficient at eliminating the U87vIII cells than CAR-F263. These response profiles support observations described in Blomberg et al.[33], wherein CAR-F263 showed a faster and more robust U87vIII killing compared to CAR-F269 and Mock T cells. This fast and robust killing activity of CAR-F263 is consistent with the auto- and non-specific activation characteristics reported for CAR-F263 [33]. A similar effector function trend, but with an earlier onset of U87vIII killing activity, was observed when adding the CAR-T/T cells to empty (no iBEC) inserts (Fig. 3b, d). The cytotoxicity kinetics are proportional to the number of CAR-T/T cells detected in the abluminal chamber over time (Fig. 3e–g). Taken together, these results suggest that the presence of the iBECs may delay the crossing of the CAR-T/T cells to the abluminal compartment (Fig. 3e), reflecting the earlier onset of U87vIII killing in the empty insert conditions (Fig. 3a vs Fig. 3b). Real-time images, acquired using Incucyte, of U87vIII cytotoxicity in the companion plates is shown in Additional file 3: Movie S1.

### Visualization of CAR-T extravasation

**Fig. 4:**
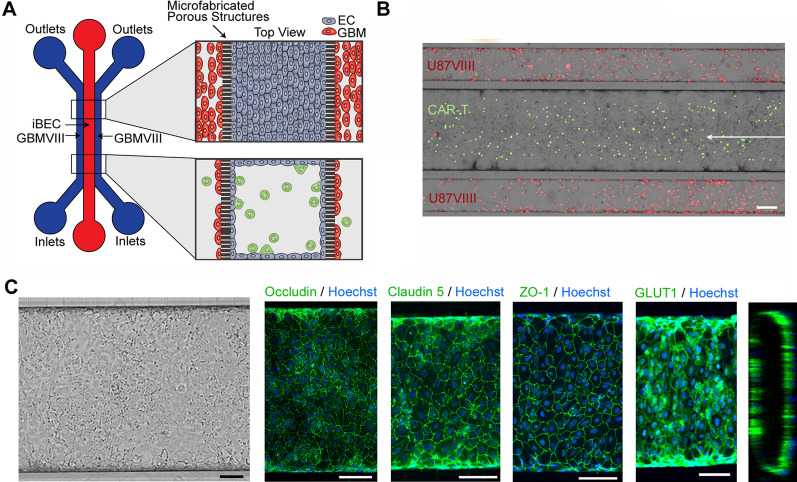
Establishment of blood–brain-tumor-barrier (BBTB) chip. **A** Schematic illustrating set up of BBB-on-CHIP systems to establish the blood–brain-tumor barrier (BBTB) model using the SynBB parallel three channel (200:500:200 µm) configuration separated by 3 µm microfabricated pores. **B** Immunofluorescence image of the BBTB model setup in the SynBBB CHIPs with iBECs seeded in the middle (endothelial) channel and U87vIII-mKate2 cells (red) seeded in the two outer channels. CAR-F263 (green) perfusion can be seen within the iBEC channel; arrow indicating flow direction (see also Additional file 3: Movie S2). Scale bar = 100 µm. **C** Phase contrast image of iBECs seeded in the centre channel and fluorescence staining for tight junction proteins OCCLUDIN, CLAUDIN 5, ZO-1 s and GLUT1 (all green) showing continuous membrane expression, cobblestone morphology and 3D lumen formation. Hoechst counterstain (blue). Scale bar = 100 µm

**Fig. 5:**
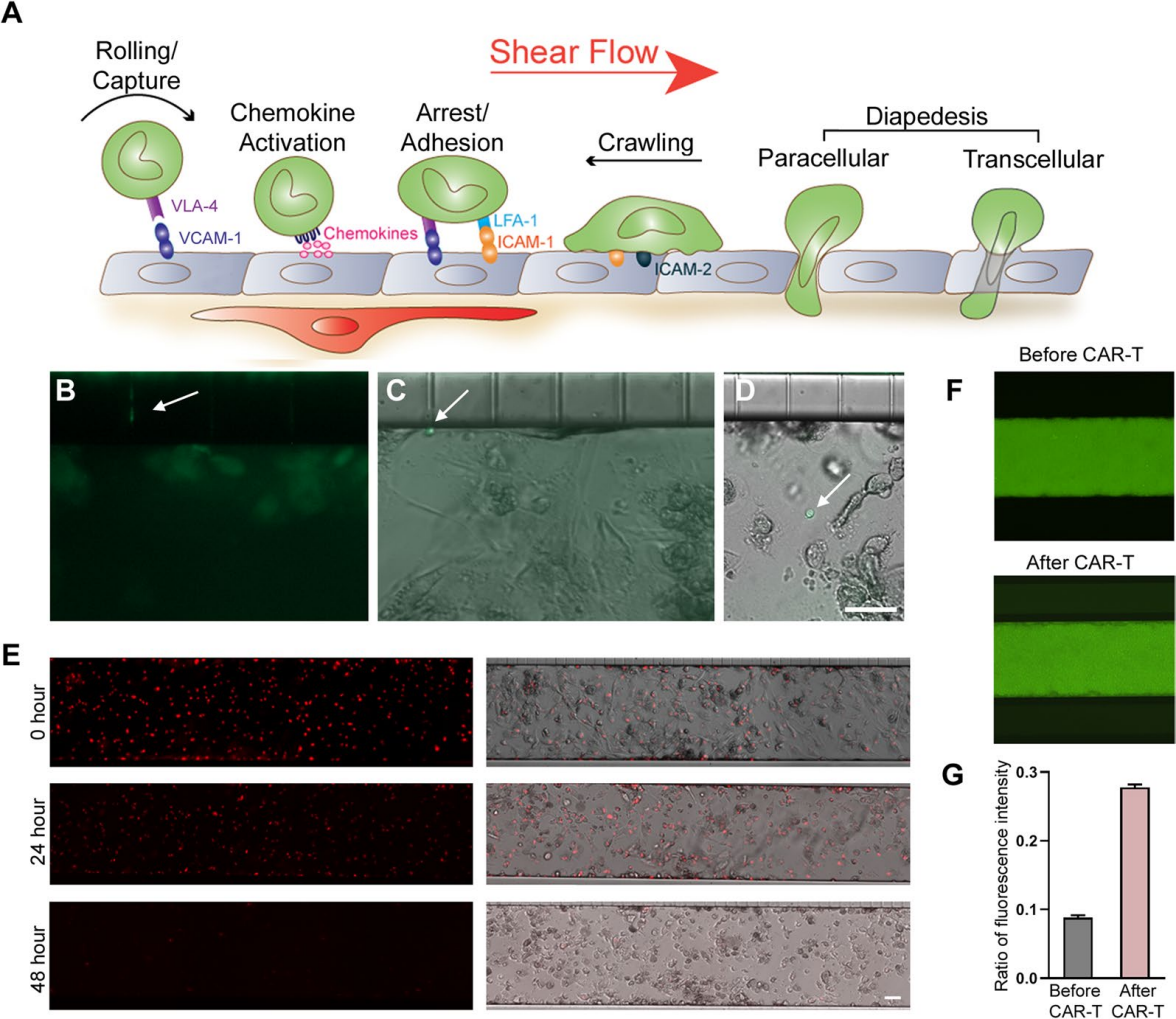
CAR-F263 extravasation and U87vIII cytotoxicity in BTBB-on-chip model. **A** Schematic illustrating sequence of immune cell adhesion and extravasation across the BEC monolayers in the presence of shear stress. **B** Image of CAR-F263, labeled with CytoLight Rapid Green Reagent, extravasation through the pores of the SynBBB chips. Arrow: CAR-F263 traversing through 3 µm pore. **C–D** Visualization of CAR-F263 in the of U87vIII channel post-extravasation. Scale bar = 50 µm (**E**) Representative images of U87vIII-mKate2 cells (red), pre (0 h) and post (24 and 48 h) perfusion of CAR-F263. U87vIII-mKate2 cell death is observed at 48 h post-CAR-F263 perfusion indicating post BBB cytotoxicity. Scale bar = 100 µm. **F** Perfusion of the middle iBEC channel with sodium fluorescein (NaFl–green) confirming intact BBB barrier integrity (before CAR-F263 perfusion) and evidence of barrier disruption (leakiness) after CAR-F263 perfusion. Evidence of BBB disruption is visualized by NaFl signal leaking into the adjacent channels. **G** Assessment of barrier integrity by quantification of average NaFl ratio intensity in the outer to inner channels using ImageJ (mean + SD)

Immune cell trafficking across the BBB is a multistep process regulated by the sequential interaction between endothelial and immune cells which is influenced by shear stress [49–51]—a key parameter lacking in conventional transwell BBTB models. Shear stress has been shown to further induce higher TEER, upregulate tight junction proteins and BBB transporters, as well as increase expression of cell adhesion molecules [49–55]. To better visualize immune cell trafficking across the BBB, we used the SynBBB microfluidic chips [56–58] to establish a 3D microfluidic based BBTB model [59, 60]. The SynBBB chips are composed of parallel vascular and tissue channels (200:500:200 µm configuration) separated by 3 µm microfabricated pores (Fig. 4a). We seeded iBEC into the center channel of the SynBBB chip with U87vIII co-cultures seeded in the adjacent channels (Fig. 4b). The iBECs formed perfusable hollow 3D lumens within the inner channel and expressed key tight junction proteins OCCLUDIN, CLAUDIN 5, ZO-1 as well as GLUT1 (Fig. 4c). For these experiments, we focused on the more tonically active CAR-F263 cells. Thus, we perfused CAR-F263 cells using flow conditions and then recorded real-time movies to examine immune cell adhesion and transmigration into the adjacent U87vIII channels (Fig. 4b, Additional file 3: Movie S2). We were able to visualize clear examples of the arrest and formation of adhesive interactions between CAR-F263/T cells and iBECs (Additional file 3: Movie S3a and 4) as well as transendothelial migration through the channel pores (Fig. 5a–d, Additional file 3: Movie S3a–b). We observed changes in T cell morphology, toward a pro-migratory phenotype, within the pores of the microfluidic device (Fig. 5b, Additional file 3: Movie S3a, b) and identified CAR-F263 cells in the adjacent U87vIII channel (Fig. 5c, d). Robust CAR-F263 mediated cytotoxicity of U87vIII was observed in the adjacent channels over the 48 h culture period, recapitulating the killing profile and kinetics observed in the transwell assays (Fig. 5e, Fig. 3a). In order to assess BBB integrity, we perfused the iBEC lumen channel with NaFl before (Fig. 5f, top) and after perfusion with CAR-F263 cells (Fig. 5f, bottom) and observed an increase in NaFl permeability after 24 h perfusion of CAR-F263 cells (Fig. 5g). These findings are in agreement with the observations in the transwell cultures (Fig. 2c) substantiating how examining alterations in BBB permeability can be a predictor of iBEC off-target toxicity or auto-activation profiles of certain CAR-T cells.

## Discussion

Systemic administration of GBM-targeting CAR-T immunotherapies are faced with unique challenges. Their access to the brain tumor is impeded by the BBB and BBTB. Furthermore, undesired crossing of B-cell targeted CAR-T cells into the brain is a driver of ICANS [6], which may also be present in systemically-administered CAR-Ts for solid tumors. Therefore, in vitro models to study the interaction of CAR-T cells with the BBB are critically needed. In this study, we leveraged human iPSC-derived brain endothelial like cells (iBECs) transwell and microfluidic BBB-on-CHIP (SynBBB) models to assess EGFRvIII-targeted CAR-T extravasation across the BBB, to validate post-BBB effector function on target cancer cells (U87vIII) and to assess accompanying neurotoxicity related to the disruption of the BBB.

iPSC-derived iBECs have been an important model for advancing human BBB permeability and drug delivery studies (ranging from small molecules to biologics), for elucidating BBB dysfunction in neurological disorders and for understanding brain susceptibility to neurotropic viruses (reviewed in [61]). Based on an extensive transcriptomic analyses [62], the iBECs phenotype has been described recently as more epithelial-like. Nevertheless, in vitro models using these cells typically exhibit strong functional barrier properties and also express multiple BBB specific receptors, transporters and efflux pumps; important criteria for studying barrier regulation and drug delivery applications in the CNS.

In this study, we used iBECs to assess cytokine-induced endothelial cell activation and concomitant expression of immune cell adhesion molecules under inflammatory conditions. Stimulation of iBECs with pro-inflammatory cytokines and U87vIII co-cultures, induced expression of ICAM-1 and VCAM-1, but not other immune adhesion molecules such as VCAM-2, S- and P-Selectin; similar to what was previously reported for other iPSC-derived BECs [40, 42, 63]. Recent efforts to improve differentiation strategies to generate iBECs with a more robust endothelial and adhesion molecule phenotype have been described [42, 64]; however, these models lacked high TEER barrier properties characteristic of the BBB. Since inflammatory induced expression of VCAM-1 and ICAM-1 and sufficient barrier properties are a prerequisite for evaluating CAR-T/T cell mediated mechanisms involved in T cell diapedesis across the BBB [65], we used this model to assess CAR-T extravasation across the iBEC monolayer. Immune cell extravasation across the BBB is a multistep process that is regulated by the sequential interaction of different signaling and adhesion molecules on the endothelial and immune cells [66]. To re-create flow-based in vivo conditions, where these interactions occur under shear stress, we used a microfluidic based BBTB-on-CHIP model. Flow-derived shear forces generate mechanical stimuli that work in concert with biochemical signals to modulate leukocyte–endothelial cell interactions, increasing the probability of leukocyte engagement of their chemokine receptors, facilitating integrin activation and consequent arrest [51]. Furthermore, these BBTB models also facilitate recapitulating the complexity of the GBM tumor microenvironment that is recognized as highly immunosuppressive [67]. As such, the immune microenvironment poses a major hurdle for CAR-T trafficking, infiltration, persistence and proliferation limiting anti-tumor activity. Leveraging these BBTB-on-CHIP models to recapitulate the heterogeneity and immunosuppressive microenvironment of GBM will further advance the development of novel immunotherapeutic strategies [67–70].

Systemically administered CAR-T therapies targeting CNS tumors will need to cross the BBTB in order to reach the tumor site, although the possibility of extravasation and migration via the choroid plexus cannot be excluded [71]. In this study, we used both the transwell and BBB-on-CHIP systems to examine CAR-T extravasation across the BBB in U87vIII co-culture systems. Brain tumors are known to compromise the integrity of the BBB, resulting in a vasculature known as the BBTB, which is highly heterogeneous and characterized by numerous distinct features, including non-uniform permeability and active efflux of molecules [72]. iBEC and U87vIII co-cultures resulted in an upregulation of ICAM-1 and VCAM-1, recapitulating neuroinflammatory conditions characterized by endothelial activation, as well as increased BBB permeability. This, in turn, facilitated CAR-T cell extravasation across the BBB; the observed extravasation was at similar levels observed for immune cells in other transwell BBB models [65, 73–76]. However, both CAR-F263 and CAR-F269 caused an increase in NaFl permeability compared to Mock T cells and iBEC controls. The tonically active CAR-F263 showed the highest percentage of extravasation in the abluminal compartment, the highest level of activation and subsequently the most robust killing of the U87vIII cells. Consistent with the delayed activation of CAR-F269, these cells showed similar extravasation rates as well as killing of U87vIII cells as unmodified Mock T cells [33].

Curiously, both CAR-F263 and CAR-F269 activation, higher than that of Mock T cells, was also observed in the upper luminal chamber, which may contribute to the increased BBB permeability for CAR-Ts. The mechanisms of this CAR activation are not clear. However, it could be the consequence of endothelial-produced secreted mediators or even those secreted by U87vIII co-cultured cells passing from the abluminal chamber. This ‘basal’ CAR-T activation in the presence of iBEC –U87vIII co-cultures could explain the observed disruption in barrier tightness. Since BECs have been shown to express MHC II and the co-stimulatory molecules CD40 and ICOSL following cytokine/inflammatory stimulation [77–79], iBECs could hypothetically act as antigen presenting cells to allogeneic T cells resulting in the activation of the T cells. To confirm this hypothesis, HLA matched cultures will be needed in future studies.

Post-BBB extravasation, CAR-F263 showed a robust effector function resulting in high cytotoxicity towards target U87vIII cells, compared to Mock T cells. This ‘positive control’ then facilitated the identification of high and low responder CAR constructs [33] against CNS targets. These data indicate that CAR-F263 may be more likely to have clinical activity if administered intravenously than the non-auto activating CAR-F269. Collectively, we were able to validate CAR-T cell extravasation across the BBB, cytotoxicity on target cells and examine changes in barrier disruption for different CAR-T constructs as potential predictors of the neurotoxicity-related events.

The two serious toxicities associated with CAR-T cell therapy include CRS and ICANS. Vascular endothelial activation, resulting from the high levels of inflammatory cytokines (IL6, TNFγ and TNFβ), has been suggested to contribute to the development of CRS and ICANS after CAR-T therapy. The accompanying BBB disruption, increased permeably and influx of inflammatory cytokines and immune cells into the CNS could initiate a feedback loop of continued endothelial activation resulting in encephalopathy syndrome. Although the pivotal role of endothelial cells in CAR-T therapy-associated CRS and ICANS has been recognized, the mechanisms of CAR-T therapy-induced endothelial dysfunction as well as potential therapeutic strategies have not yet been well studied. This study demonstrates how iPSC-derived human BBB models in vitro could be of significant value as preclinical models for understanding CAR-T related neurotoxicity, as well as enabling preclinical screening and/or clinical titrations of CAR-T candidates based on BEC-induced toxicity. Collectively, these models become key in supporting the development of systemically-delivered CAR-T designs that target brain tumors [6]. Leveraging human iPSC-derived isogeneic culture of the neurovascular unit (astrocytes, pericytes and neurons) could further advance our understanding of CAR-T mediated neurotoxicity.

While these initial studies are meant as a technical proof of concept primarily, we have observed that tonic-signaling CARs exhibit a higher level of BBB transmigration, perhaps suggesting that CAR molecules developed for peripheral cancers, with lower tonic activity, might also show lower neurotoxicity. Furthermore, these models can also be useful in evaluating vasculo- and neuroprotective management and treatment strategies to minimize the neurotoxic effects of CAR-T therapies. The mitigation of on-target, off-tumor effects, neurotoxicity, and the potential of CRS/ICANS remain essential considerations in the development of novel CAR-T cell therapies [80, 81].

## Supplementary Information

**Additional file 1:** **Figure S1.** iBEC activation following TNFα treatment. **A** Cell surface analysis for expression of adhesion molecule VCAM-1 in iBECs using flow cytometry under non-stimulated (red) and cytokine stimulated (blue) conditions using different TNFα concentrations. **B** Validation of VCAM-1 expression in iBEC following treatment with 300ng/ml of TNFα. Hoechst counterstain (blue). Scale bar = 20 µm.

**Additional file 2:** **Figure S2.** Confirmation of CAR-T/T cell mediated iBEC cytotoxicity. **A** Staining of co-cultures of iBECs with CAR-F263, CAR-F269 and Mock T cells and iBEC alone with CellTrackerGreen (green) and Ethidium Homodimer 1 (red) after 24 hr culture. Scale bar = 400 µm (**B**) Higher magnification images showing CAR-T cells (arrow) in the iBEC cultures. Scale car = 200 µm. **C** Quantification of red object count (ethidium homodimer 1) relative to iBEC alone cultures using Incucyte as a measure of CAR-T/T mediated iBEC cell death/cytotoxicity. Relative red object count is expressed as the mean + SD. No statistical significance as assessed by one-way analysis of variance (ANOVA) by comparison to iBEC alone, where ns=P>0.05 (n=3).

**Additional file 3:** **Movie S1.** Real-time post-BBB CAR-T/T cell mediated in U87vIII killing. Real-time movies of post-BBB extravasation of CAR-F263 and CAR-F269 mediated killing of U87vIII-mKate2 cells acquired via Incucyte. No T cell is shown as a control of U87vIII proliferation in the absence of anti-EGFRvIII-CAR-targeted killing. Movies are shown over a 48 h time course. Scale bar = 200 µm. **Movie S2.** Set-up of blood-brain-tumor barrier (BBTB)-on-CHIP model system using SynBBB. Real-time movie showing immunofluorescence images of the SynBBB blood-brain-tumor-barrier setup in the SynBBB chips with iBECs seeded in the middle channel (phase contrast image) and U87vIII-mKate2 cells (red) seeded in the two outer channels. CAR-F263 (green) perfusion can be seen within the middle iBEC channel; arrow indicating perfusion direction. Movie is played at 4 frames per second. Scale bar = 100 µm. **Movie S3.** Real-time movies of T cell interaction and extravasation across the middle iBEC channel. **A-B **Real-time phase contrast images showing evidence of T cell arrest and adhesion to the iBEC monolayer in the endothelial channel and extravasation across the 3 µm microfabricated pores. Movie is played at 4 frames per second. Scale bar = 50 µm. **Movie S4.** Real-time movies of CAR-F263 cell arrest and adhesion to iBECs. Real-time phase contrast images showing evidence of CAR-F263 cell arrest and adhesion to the iBEC monolayer in the middle channel. CAR-F263 labelled with CytoLight dye (green). Movie is played at 4 frames per second.

**Additional file 4: Table S1. **Detailed information about antibodies used in the study. Table S2. Median Fluorescence Intensity (MFI) of immune cell adhesion molecules with and without stimulation.

## Data Availability

The datasets generated during and/or analyzed during the current study are available from the corresponding author upon request.
