# Supplementary figures and images for: Application of blood brain barrier models in pre-clinical assessment of glioblastoma-targeting CAR-T based immunotherapies
Source: Fluids Barriers CNS. 2022 Jun 1;19:38. doi: 10.1186/s12987-022-00342-y (PMC9161615; doi:10.1186/s12987-022-00342-y)

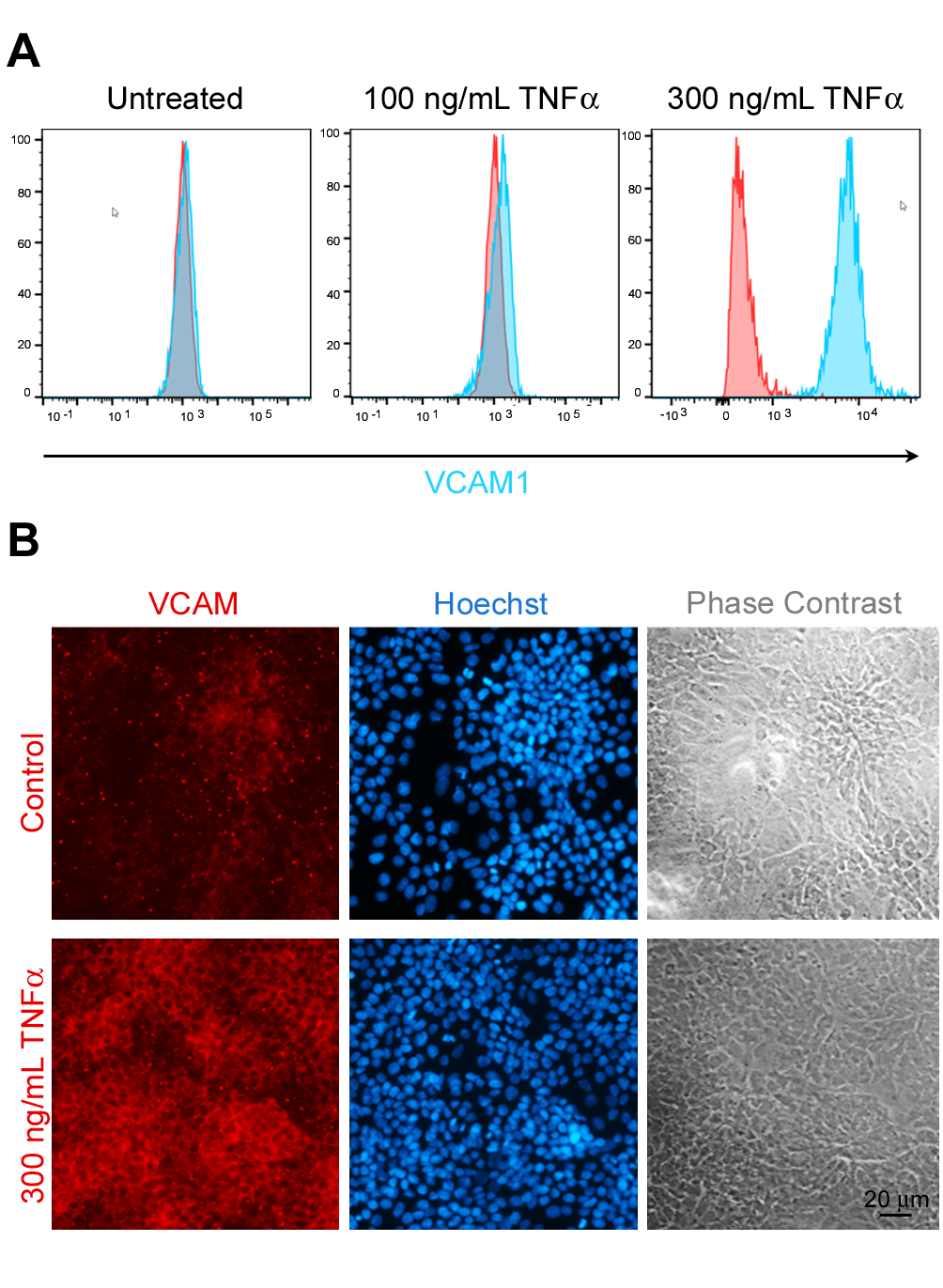

Supplement: Supplementary file 1 — Additional file 1: Figure S1. iBEC activation following TNFα treatment. A Cell surface analysis for expression of adhesion molecule VCAM-1 in iBECs using flow cytometry under non-stimulated (red) and cytokine stimulated (blue) conditions using different TNFα concentrations. B Validation of VCAM-1 expression in iBEC following treatment with 300ng/ml of TNFα. Hoechst counterstain (blue). Scale bar = 20 µm. [file 12987_2022_342_MOESM1_ESM.tif]

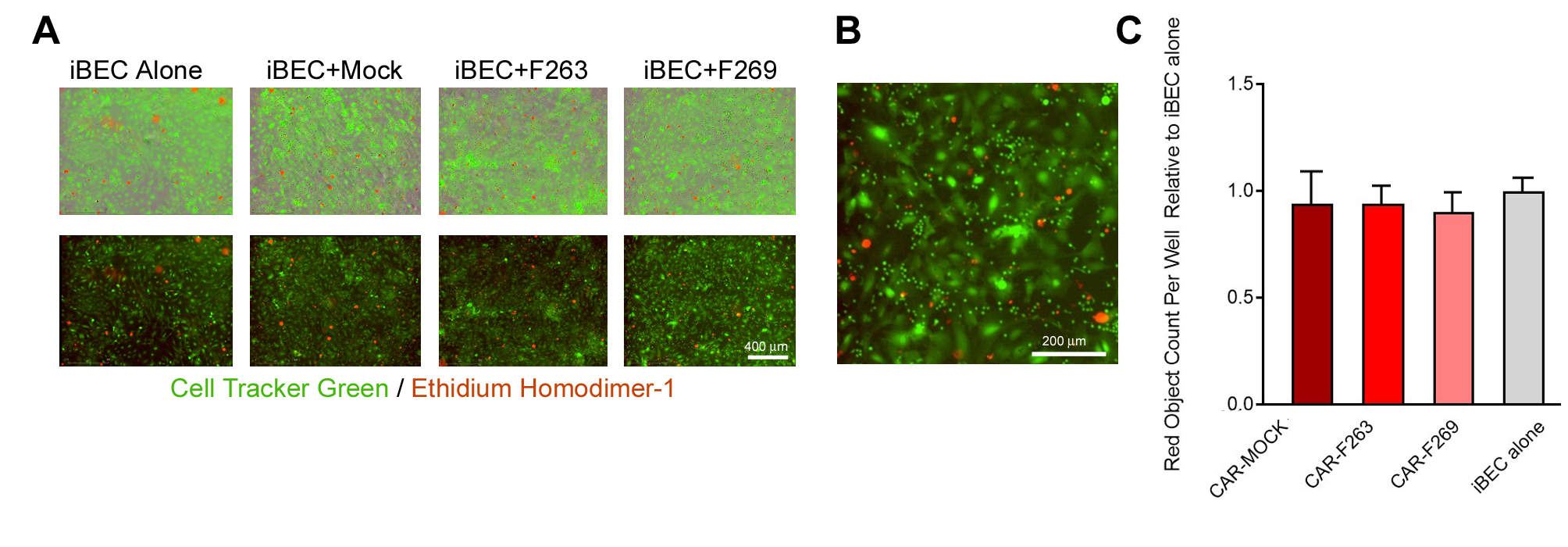

Supplement: Supplementary file 2 — Additional file 2: Figure S2. Confirmation of CAR-T/T cell mediated iBEC cytotoxicity. A Staining of co-cultures of iBECs with CAR-F263, CAR-F269 and Mock T cells and iBEC alone with CellTrackerGreen (green) and Ethidium Homodimer 1 (red) after 24 hr culture. Scale bar = 400 µm (B) Higher magnification images showing CAR-T cells (arrow) in the iBEC cultures. Scale car = 200 µm. C Quantification of red object count (ethidium homodimer 1) relative to iBEC alone cultures using Incucyte as a measure of CAR-T/T mediated iBEC cell death/cytotoxicity. Relative red object count is expressed as the mean + SD. No statistical significance as assessed by one-way analysis of variance (ANOVA) by comparison to iBEC alone, where ns=P>0.05 (n=3). [file 12987_2022_342_MOESM2_ESM.tif]
