# Supplementary material for: Application of blood brain barrier models in pre-clinical assessment of glioblastoma-targeting CAR-T based immunotherapies
Source: Fluids Barriers CNS. 2022 Jun 1;19:38. doi: 10.1186/s12987-022-00342-y (PMC9161615; doi:10.1186/s12987-022-00342-y)
Supplement: Supplementary file 3 — Additional file 3: Movie S1. Real-time post-BBB CAR-T/T cell mediated in U87vIII killing. Real-time movies of post-BBB extravasation of CAR-F263 and CAR-F269 mediated killing of U87vIII-mKate2 cells acquired via Incucyte. No T cell is shown as a control of U87vIII proliferation in the absence of anti-EGFRvIII-CAR-targeted killing. Movies are shown over a 48 h time course. Scale bar = 200 µm. Movie S2. Set-up of blood-brain-tumor barrier (BBTB)-on-CHIP model system using SynBBB. Real-time movie showing immunofluorescence images of the SynBBB blood-brain-tumor-barrier setup in the SynBBB chips with iBECs seeded in the middle channel (phase contrast image) and U87vIII-mKate2 cells (red) seeded in the two outer channels. CAR-F263 (green) perfusion can be seen within the middle iBEC channel; arrow indicating perfusion direction. Movie is played at 4 frames per second. Scale bar = 100 µm. Movie S3. Real-time movies of T cell interaction and extravasation across the middle iBEC channel. A-B Real-time phase contrast images showing evidence of T cell arrest and adhesion to the iBEC monolayer in the endothelial channel and extravasation across the 3 µm microfabricated pores. Movie is played at 4 frames per second. Scale bar = 50 µm. Movie S4. Real-time movies of CAR-F263 cell arrest and adhesion to iBECs. Real-time phase contrast images showing evidence of CAR-F263 cell arrest and adhesion to the iBEC monolayer in the middle channel. CAR-F263 labelled with CytoLight dye (green). Movie is played at 4 frames per second. [file 12987_2022_342_MOESM3_ESM.pptx]

## Slide 1
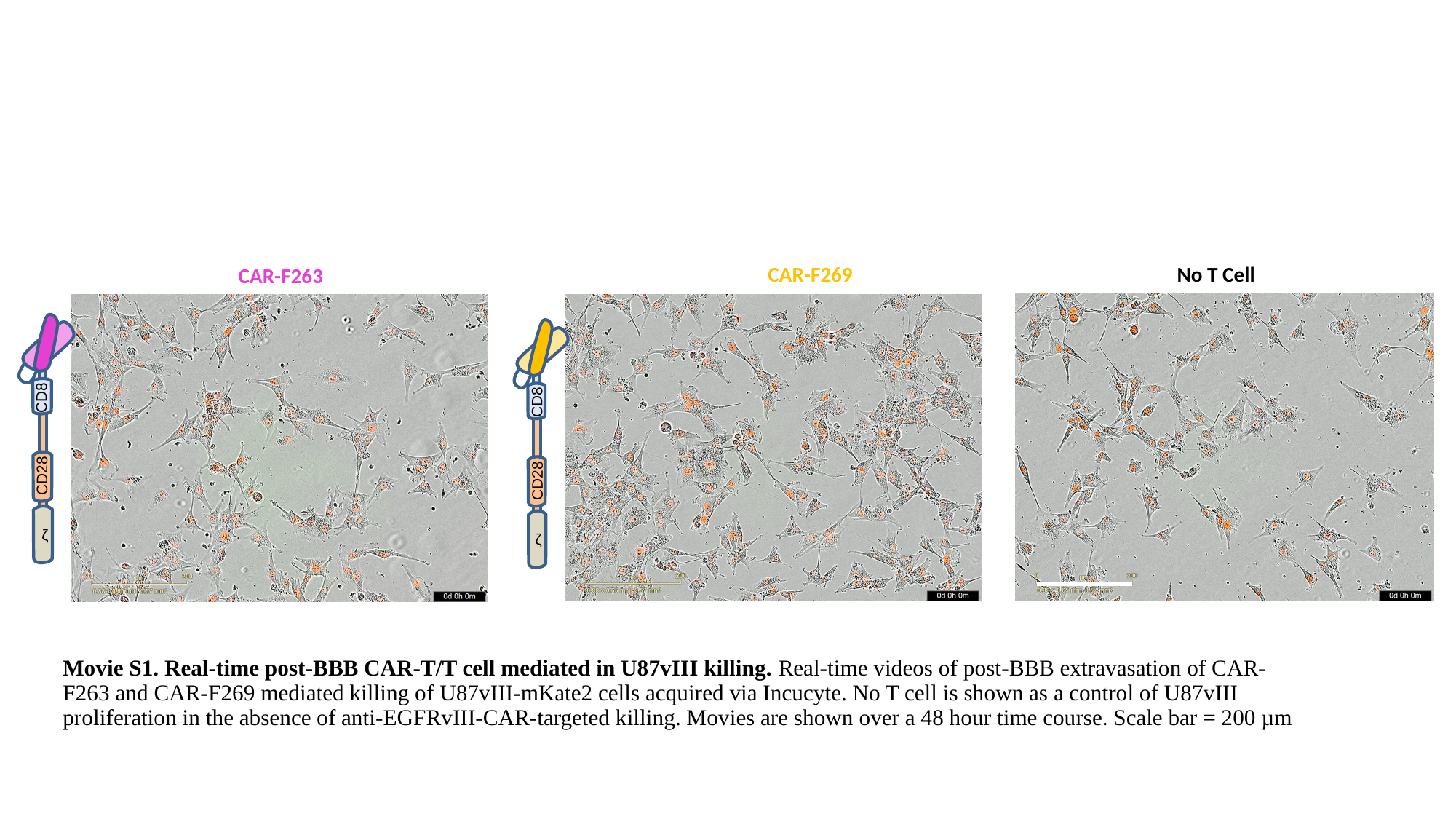

CAR-F269
CD8
CD28
ζ
No T Cell
CAR-F263
CD8
CD28
ζ
# Movie S1. Real-time post-BBB CAR-T/T cell mediated in U87vIII killing. Real-time videos of post-BBB extravasation of CAR-F263 and CAR-F269 mediated killing of U87vIII-mKate2 cells acquired via Incucyte. No T cell is shown as a control of U87vIII proliferation in the absence of anti-EGFRvIII-CAR-targeted killing. Movies are shown over a 48 hour time course. Scale bar = 200 µm

## Slide 2
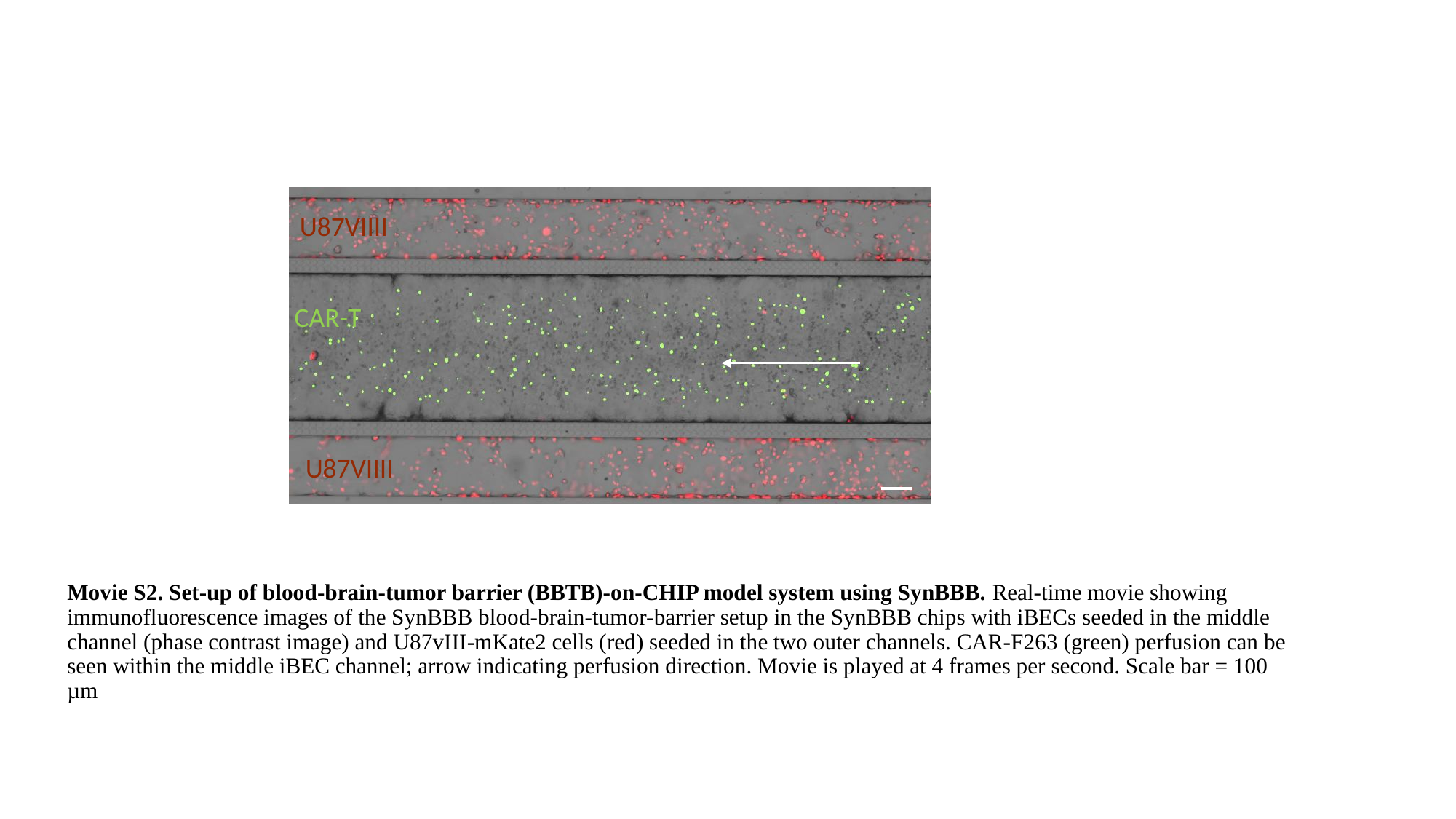

U87VIIII
CAR-T
U87VIIII
# Movie S2. Set-up of blood-brain-tumor barrier (BBTB)-on-CHIP model system using SynBBB. Real-time movie showing immunofluorescence images of the SynBBB blood-brain-tumor-barrier setup in the SynBBB chips with iBECs seeded in the middle channel (phase contrast image) and U87vIII-mKate2 cells (red) seeded in the two outer channels. CAR-F263 (green) perfusion can be seen within the middle iBEC channel; arrow indicating perfusion direction. Movie is played at 4 frames per second. Scale bar = 100 µm
Before CAR-T

## Slide 3
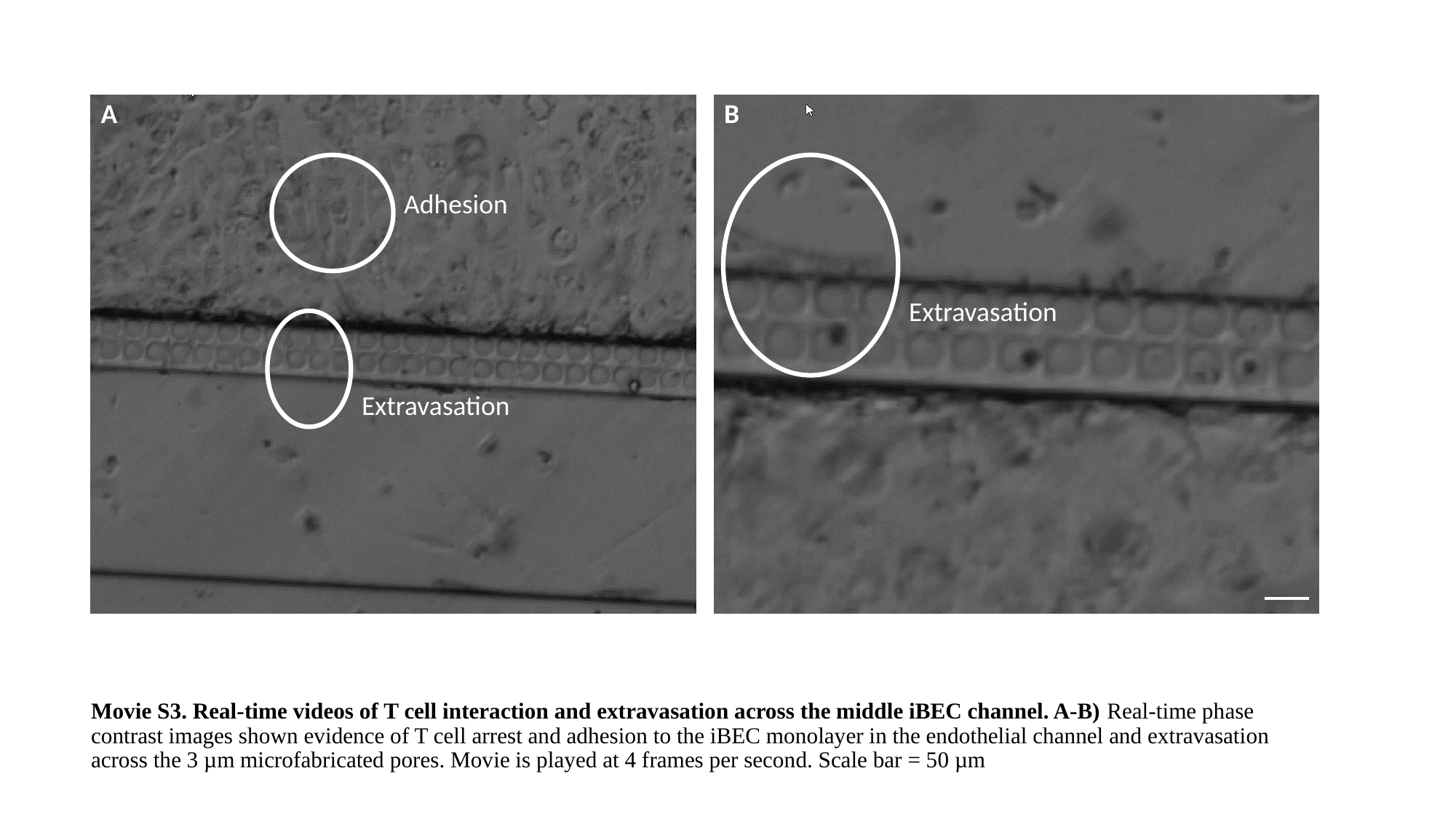

A
B
Adhesion
Extravasation
Extravasation
# Movie S3. Real-time videos of T cell interaction and extravasation across the middle iBEC channel. A-B) Real-time phase contrast images shown evidence of T cell arrest and adhesion to the iBEC monolayer in the endothelial channel and extravasation across the 3 µm microfabricated pores. Movie is played at 4 frames per second. Scale bar = 50 µm

## Slide 4
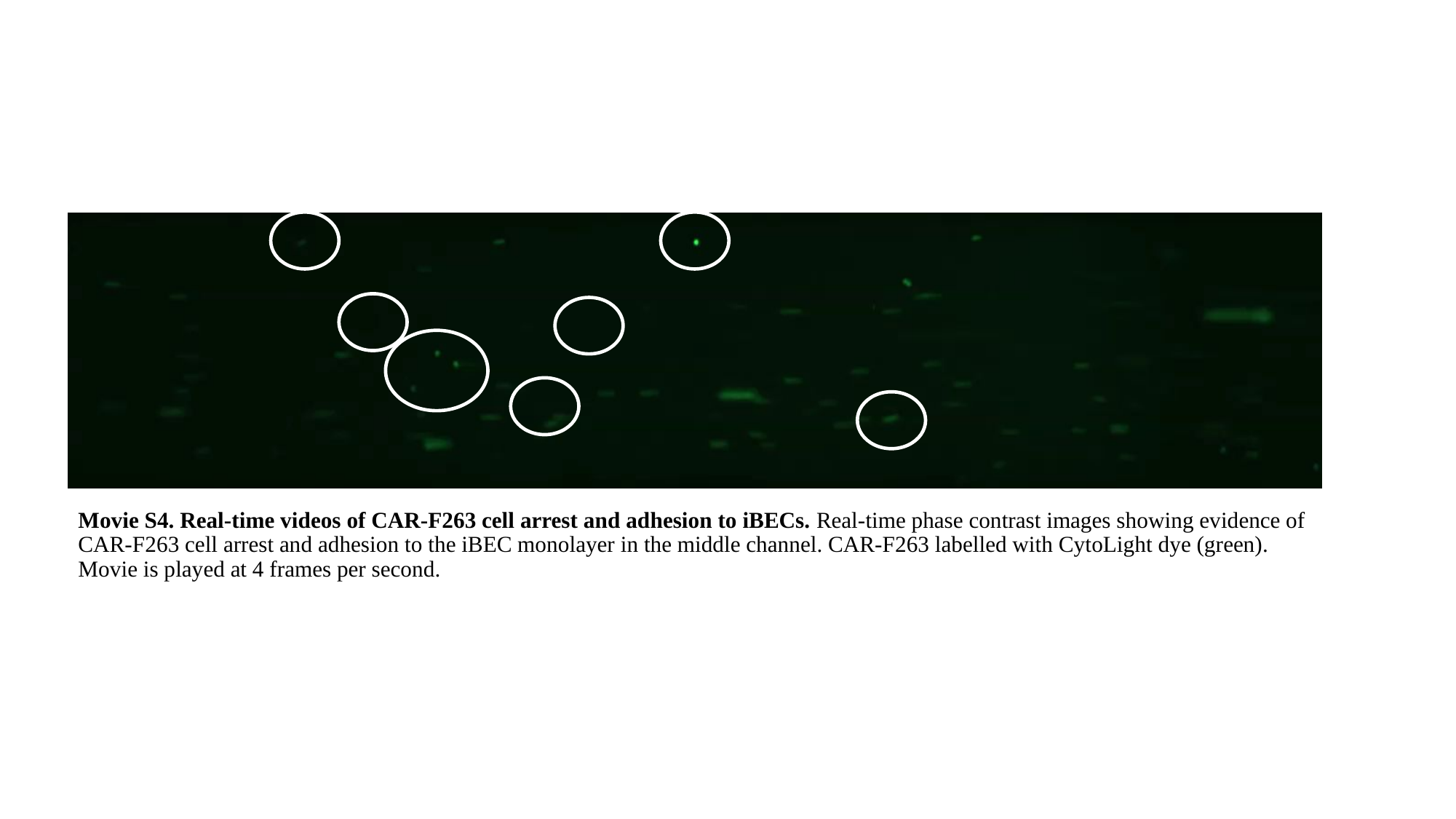

# Movie S4. Real-time videos of CAR-F263 cell arrest and adhesion to iBECs. Real-time phase contrast images showing evidence of CAR-F263 cell arrest and adhesion to the iBEC monolayer in the middle channel. CAR-F263 labelled with CytoLight dye (green). Movie is played at 4 frames per second.
