# Supplementary material for: Application of blood brain barrier models in pre-clinical assessment of glioblastoma-targeting CAR-T based immunotherapies
Source: Fluids Barriers CNS. 2022 Jun 1;19:38. doi: 10.1186/s12987-022-00342-y (PMC9161615; doi:10.1186/s12987-022-00342-y)
Supplement: Supplementary file 4 — Additional file 4: Table S1. Detailed information about antibodies used in the study. Table S2. Median Fluorescence Intensity (MFI) of immune cell adhesion molecules with and without stimulation. [file 12987_2022_342_MOESM4_ESM.docx]

Additional file 4

**Table S1**

| **Antibodies** | **Vender** | **Cat#** | **Dilutions** |
| --- | --- | --- | --- |
| Immune Adhesion Markers (Flow Cytometry) | | | |
| ICAM-1 | BD Biosciences | 561899 | 1:100 |
| ICAM-2 | BD Biosciences | 558080 | 1:100 |
| P-Selectin | BD Biosciences | 550866 | 1:100 |
| E-Selection | BD Biosciences | 551144 | 1:100 |
| CD99 | BioLegend | 371313 | 1:100 |
| VCAM-1 | BD Biosciences | 551146 | 1:100 |
| CD45 | [BD](https://www.bdbiosciences.com/ca/reagents/research/antibodies-buffers/immunology-reagents/anti-mouse-antibodies/cell-surface-antigens/bv786-rat-anti-mouse-cd45ra-148/p/747759) Biosciences | 563716 | 1:750 |
| CD25 | BD Biosciences | 335824 | 1:750 |
| BBB Markers (BBB-on-CHIP Immunofluorescence | | | |
| CLAUDIN 5 | ThermoFisher | 352588 | 1:50 |
| OCCLUDIN | ThermoFisher | 331588 | 1:100 |
| ZO-1 | ThermoFisher | 339188 | 1:100 |
| GLUT-1 | Abcam | 195359 | 1:50 |

|  |  | **MFI** | | |
| --- | --- | --- | --- | --- |
|  | Surface Makers | Unstained | Non-stimulated | Stimulated |
| **Cytokine**  **Stimulated** | ICAM-1 | 296 | 1753 | 5142 |
|  | ICAM-2 | 787 | 1172 | 1180 |
|  | P-selectin | 1909 | 2273 | 2568 |
|  | E-selectin | 496 | 525 | 564 |
|  | CD99 | 1408 | 15270 | 1520 |
|  | VCAM-1 | 787 | 847 | 68068 |
| **GBM**  **co-cultures** | ICAM-1 | 1431 | 2568 | 3146 |
|  | ICAM-2 | 700 | 1152 | 1055 |
|  | P-selectin | 1431 | 2568 | 3146 |
|  | E-selectin | 480 | 723 | 723 |
|  | CD99 | 1072 | 15520 | 11352 |
|  | VCAM-1 | 847 | 981 | 28196 |

**Table S2**
